# Epidemiological Characteristics of Varicella Outbreaks in Daegu and Gyeongsangbuk-do, the Republic of Korea, 2022–2025

**DOI:** 10.56786/PHWR.2026.19.40.1

**Published:** 2026-10-22

**Authors:** Seong Hee Oh, Minjei Lee, Sookhyun Kim, Myung-Jae Hwang, Sang Yun Cho

**Affiliations:** 1 Division of Immunization Services, Department of Healthcare Safety and Immunization, Korea Disease Control and Prevention Agency Cheongju Korea; 2 Division of Infectious Disease Control and Response, Gyeongbuk Regional Center for Disease Control and Prevention, Korea Disease Control and Prevention Agency Daegu Korea

**Keywords:** Chickenpox, Disease outbreaks, Schools, Vaccination

## Abstract

**Objectives:**

This study aimed to characterize the epidemiological patterns of varicella outbreaks in Daegu and Gyeongsangbuk-do from 2022 to 2025.

**Methods:**

Varicella outbreaks reported through the Korea Disease Control and Prevention Agency surveillance system in Daegu and Gyeongsangbuk-do from January 2022 through December 2025 were analyzed. Of the 235 reported outbreaks, 213 met the inclusion criteria after excluding reclassified, duplicate, deleted, and out-of-region outbreaks. Outbreak size and duration were analyzed for 209 outbreaks involving 2,253 outbreak-associated cases with available case-level information.

**Results:**

The number of varicella outbreaks increased from 14 in 2022 to 84 in 2024, and outbreak-associated cases also peaked in 2024, at 898. Elementary schools accounted for 59.6% of outbreaks, and most outbreaks involving 11 or more cases occurred in elementary schools. Outbreak size did not differ significantly across years, whereas outbreak duration differed significantly across years.

**Conclusions:**

This study characterized the epidemiological patterns of varicella outbreaks in Daegu and Gyeongsangbuk-do from 2022 to 2025 and found that outbreaks occurred most frequently in elementary schools, where most outbreaks involving 11 or more cases also occurred. These findings may serve as a basis to inform school-based varicella prevention and control efforts and to review future vaccination policies.

**Key messages**

**① What is known previously?**

A national analysis from 2018 to 2020 showed that varicella outbreaks occurred most frequently in elementary schools, with larger outbreaks and longer durations than those observed in childcare centers and kindergartens.

**② What new information is presented?**

The number of varicella outbreaks in Daegu and Gyeongsangbuk-do increased until 2024 and decreased thereafter in 2025. Outbreak size did not differ significantly by year, whereas outbreak duration showed significant differences across years.

**③ What are implications?**

This study identified the epidemiological characteristics of varicella outbreaks in Daegu and Gyeongsangbuk-do from 2022 to 2025. These findings may serve as a basis to inform school-based prevention and control efforts and to review future vaccination policies.

## Introduction

Varicella is an acute exanthematous infectious disease caused by the varicella-zoster virus and is an infectious disease transmitted only by humans, without other vectors such as animals or insects. It is transmitted through direct contact with the vesicular lesions of a patient with varicella, or by inhaling aerosols generated from the vesicle fluid of skin lesions or respiratory secretions of a patient with acute varicella or herpes zoster [1]. Because it is highly infectious, when there is a varicella case within a household, the secondary attack rate among susceptible household contacts is reported to be 80–90%, and the infectious period lasts from 1–2 days before the onset of rash until crusts form on all lesions [2]. The incubation period of varicella is 10–21 days after exposure, and the average incubation period is 14–16 days. The clinical manifestations of varicella progress rapidly within 24 hours, accompanied by fever, in the order of macules, papules, vesicles, pustules, and crusts. In healthy children, symptoms are mild and systemic symptoms improve within 2–4 days of rash onset, while in children with reduced immunity or adults, the incidence of complications, such as secondary infection of the rash site, Reye syndrome, encephalitis, and pneumonia, may be higher. In addition, if a pregnant woman within 20 weeks of gestation is infected with primary varicella, congenital varicella syndrome associated with neonatal malformations may occur [1].

Varicella is a representative notifiable infectious disease that mainly occurs among school-aged children, with approximately 80,000–90,000 cases reported annually prior to the coronavirus disease 2019 (COVID-19) pandemic [3]. In addition, it exhibits seasonal characteristics with an increase in occurrence during the school terms of April–July and November–December [1].

The Republic of Korea (ROK) has included varicella in the National Immunization Program (NIP) since 2005, and recommends one dose at 12–15 months of age for all children [4]. During 2020–2022, the varicella vaccination coverage among children aged 2 years was reported to be 96% or higher [5-7], and the effectiveness of the varicella vaccine has been reported to be approximately 79% with a single dose [8].

According to data from the Korea Disease Control and Prevention Agency (KDCA) Infectious Disease Portal, some respiratory infectious diseases, such as varicella, mumps, and pertussis, decreased during the COVID-19 pandemic and have shown a tendency to gradually increase since 2022 [9]. Since 2022, domestic studies analyzing the outbreak size and outbreak duration of local-level varicella outbreaks have been limited. Therefore, using data on varicella outbreaks in the Gyeongbuk region (Daegu Metropolitan City, Gyeongsangbuk-do) reported to the Infectious Disease Information System (IDIS) from 2022 to 2025, we analyzed the occurrence patterns and epidemiological characteristics of varicella outbreaks in the Gyeongbuk region in the post COVID-19 period.

## Methods

### 1. Study Population

The study included cases reported as varicella cases or suspected cases in the KDCA’s IDIS from January 2022 to December 2025. According to the diagnostic and reporting criteria for varicella, a case was defined as a person with clinical manifestations consistent with varicella whose infection was confirmed by laboratory diagnostic criteria, and a suspected case was defined as a person in whom varicella was suspected considering clinical manifestations and epidemiological links but who lacked test results meeting the diagnostic testing criteria [1]. For outbreaks, the outbreak criteria from the 2022–2025 KDCA Guidelines for the Management of Vaccine-Preventable Diseases were applied [10-13]. An outbreak was defined as the occurrence of two or more cases or suspected cases in the same class or facility within 3 weeks, or a situation where two or more individuals were closely linked in time and space. Among these, events classified as clusters based on epidemiological investigation results were selected as the study population. Of 235 clusters, 213 clusters were analyzed, excluding 22 clusters that were reclassified as belonging to the previous year, were duplicates, were deleted by public health centers, or were outside the jurisdiction of the Gyeongbuk region (Table 1). For cluster size and outbreak duration, the analysis was limited to clusters where cluster-related case information could be verified. A total of 209 clusters (with 2,253 cluster-related cases) were analyzed, excluding two clusters where only one case was confirmed and two clusters where case information could not be verified.

### 2. Data Collection and Management

Using KDCA’s IDIS, varicella outbreaks and individual epidemiological investigation data related to clusters reported in the Gyeongbuk region from 2022 to 2025 were initially collected as of January 30, 2026. Subsequently, to update the 2025 reporting data, the data were recollected as of July 22, 2026, to construct the final dataset.

The number of cases in each outbreak was verified by comparing the outbreak epidemiological investigation results with the individual epidemiological investigation data. In cases where the number of cases between the two sources was inconsistent, the data were corrected based on the individual epidemiological investigation data. Duplicate cases, cases deleted by public health centers, and cases outside the jurisdiction of the Gyeongbuk region were excluded, and cases corresponding to clusters from the previous year were reclassified based on the symptom onset date of the index case.

### 3. Variable Definitions

The year and month of occurrence of outbreaks were classified based on the symptom onset date of the index case within the cluster. The region of occurrence was classified by city/province where the cluster occurred, and the facility type was classified into childcare center, kindergarten, elementary school, middle and high school, and other. “Other” included factories, agricultural cooperatives, hospitals, churches, welfare facilities, rehabilitation centers, and military units. Cluster size was defined as the number of cluster-related cases. The outbreak duration extended from the symptom onset date of the index case to that of the last case within the cluster, and was calculated by adding 1 day to the difference between the two dates to include both the first and last symptom onset dates.

### 4. Analytical Methods

Differences in cluster size and outbreak duration by year and facility type were analyzed using the Kruskal–Wallis test, and if statistically significant, the Dwass–Steel–Critchlow–Fligner post hoc test was performed. Statistical analysis was performed using Jamovi (ver. 2.3.28), and Microsoft Excel 2016 was used for data aggregation, organization, and graphing. The level of statistical significance was set at p<0.05.

### 5. Use of Artificial Intelligence Tools

An artificial intelligence tool, ChatGPT (GPT-5.6, OpenAI), was used as a supplementary tool for the English translation in some parts and sentence editing of this manuscript, and the content was reviewed and revised by the author.

## Results

### 1. General Characteristics

From 2022 to 2025, a total of 213 varicella outbreaks were identified in the Gyeongbuk region. By region, there were 157 outbreaks (73.7%) in Daegu Metropolitan City and 56 outbreaks (26.3%) in Gyeongsangbuk-do, showing a higher reporting rate in Daegu Metropolitan City. By type of facility, elementary schools had the highest number with 127 outbreaks (59.6%), followed by middle and high schools with 58 outbreaks (27.2%), other with 10 outbreaks (4.7%), and childcare centers and kindergartens with 9 outbreaks (4.2%) each. By year, elementary school outbreaks were the most frequent in all years (Table 2).

### 2. Annual and Monthly Occurrence Status of Varicella Outbreaks

The annual number of varicella outbreaks was 14 in 2022, 53 in 2023, 84 in 2024, and 62 in 2025, with the highest occurrence in 2024. The number of cluster-related cases was 97 in 2022, 572 in 2023, 898 in 2024, and 686 in 2025. The months with the highest monthly number of cluster occurrences were November 2022, March 2023, May 2024, and April 2025, with 5, 9, 18, and 13 clusters, respectively. The months with the highest monthly number of cluster-related cases were November 2022, December 2023, April 2024, and April 2025, with 31, 132, 171, and 201 cases, respectively.

The overall number of varicella cases in the Gyeongbuk region tended to increase in the spring and at the end of the year and decrease in the summer from 2023 to 2025 (Figure 1).

### 3. Occurrence Status by Region and Facility Type

By region, the number of cluster-related cases in both Daegu Metropolitan City and Gyeongsangbuk-do was the highest in 2024, with 664 and 234 cases, respectively. By type of facility, elementary schools had the highest number of cluster-related cases in all years, with 74, 409, 589, and 457 cases in 2022, 2023, 2024, and 2025, respectively. Middle and high schools had the highest number of cluster-related cases after elementary schools, with the highest number observed in 2024 at 203 cases. By cluster size, elementary schools accounted for 30 out of 49 clusters of 11–20 cases (61.2%), 17 out of 23 clusters of 21–40 cases (73.9%), and 4 out of 5 clusters of 41 or more cases (80.0%).

### 4. Cluster Size and Outbreak Duration

An analysis of 209 clusters (2,253 cluster-related cases) for cluster size and outbreak duration showed that, by cluster size, clusters of 2–10 cases were the most common with 132 clusters (63.2%), followed by 11–20, 21–40, and >40 cases with 49 (23.4%), 23 (11.0%), and 5 clusters (2.4%), respectively. By year, for 2022, 2023, 2024, and 2025, we recorded 11, 31, 55, and 35 clusters of 2–10 cases; 3, 12, 17, and 17 clusters of 11–20 cases; and 0, 1, 3, and 1 cluster of >40 cases, respectively.

The overall median cluster size was 7 cases (3.0–14.0), and by year, the sizes were 6 (3.5–8.0), 7 (4.5–12.5), 6 (3.0–15.0), and 10 cases (4.0–13.3) in 2022, 2023, 2024, and 2025, respectively, with the largest size in 2025. However, the difference in cluster size by year was not statistically significant (p=0.353).

The overall median outbreak duration for 2022–2025 was 31 days (16.0–49.0), with 23 (12.5–32.3), 32 (20.0–57.5), 23 (16.0–45.3), and 34 days (20.5–56.5) in 2022, 2023, 2024, and 2025, respectively. Thus, the median duration was the longest in 2025. In addition, the outbreak duration varied significantly by year (p=0.010) (Table 3). The post hoc test showed that the outbreak duration in 2025 was significantly longer than that in 2022 (p=0.040) and 2024 (p=0.038); however, no significant differences were observed between other years.

The median cluster sizes by type of facility were 6 (3.0–6.0), 11 (9.0–12.0), 8 (4.0–15.5), 6 (3.0–11.0), and 3 (2.0–7.0) in childcare centers, kindergartens, elementary schools, middle and high schools, and other facilities, respectively (Table 3). The cluster sizes differed significantly by type of facility (p=0.022); however, the post hoc test revealed no significant differences between individual facility types. The median outbreak duration was 17 (6.0–31.0), 27 (20.0–35.0), 33 (16.0–58.0), 30 (18.0–49.0), and 18 days (7.0–18.5) for childcare centers, kindergartens, elementary schools, middle and high schools, and other, respectively. The outbreak duration differed significantly by facility type (p=0.020); however, the post hoc test showed no significant difference between individual facility types.

## Discussion

This study analyzed 213 varicella outbreaks reported in the Gyeongbuk region from 2022 to 2025. Varicella outbreaks increased from 2022 to 2024, peaking at 84 outbreaks in 2024, and decreased to 62 outbreaks in 2025. The number of cluster-related cases also increased from 2022 to 2024, peaking at 898 cases in 2024, and decreased to 686 cases in 2025. Previous studies have reported that the incidence of varicella, which had decreased during the COVID-19 pandemic, increased again following the lifting of social distancing and the resumption of face-to-face activities [14,15]. However, this study did not include data from before the COVID-19 pandemic, presenting limitations in directly comparing occurrence patterns before and after the pandemic. Additionally, although both the number of outbreaks and cluster-related cases in 2025 decreased compared to the previous year, the study period was short at 4 years, making it difficult to determine whether this decrease is a sustained trend.

By type of facility, elementary schools were the most common, accounting for 59.6% of all clusters, and had the most cluster-related cases in all years. An analysis of varicella outbreaks during 2018–2020, which included the pre-COVID-19 pandemic period, also showed a similar pattern to this study, with elementary schools accounting for the highest proportion at 59.3% of all clusters. In addition, elementary schools have a relatively high classroom density, which might affect cluster occurrence between grades and classes due to after-school activities [2].

By cluster size, 61.2%, 73.9%, and 80.0% of clusters of 11–20, 21–40, and >40 cases occurred in elementary schools, respectively. Both cluster size and outbreak duration varied significantly by facility type; however, the post hoc test showed no significant difference between individual facility types. The median cluster size did not differ significantly by year, but the median outbreak duration differed significantly by year. The post hoc test showed that the outbreak duration in 2025 was significantly longer than the durations in 2022 and 2024. Previous domestic studies have also shown that the median outbreak duration of varicella outbreaks differed by year [16]. However, prior studies did not precisely elucidate the cause of these differences. Similarly, in the present study, identification of the factors affecting the annual differences in the outbreak duration was difficult. Thus, further analysis of related factors is warranted after the accumulation of sufficient annual data in the future.

Currently, the ROK administers a single dose of varicella vaccine to children aged 12 to 15 months as part of the NIP [4]. In the present study, outbreaks continued to occur primarily in elementary schools, and relatively large-sized outbreaks were also identified. According to previous studies, the effectiveness of the varicella vaccine is only about 79% with one dose, but has been reported to increase to about 94% with two doses [8]. It is important to determine whether the increase in varicella occurrence among elementary school-aged children occurs due to the decrease in vaccine effectiveness over time or due to the omission of the first primary dose or an increase in breakthrough infections [17]. To this end, future studies need to analyze the varicella vaccination completion rate by age while focusing on the proportion of breakthrough infections [17,18].

However, this study has several limitations. First, the study population was limited to the Gyeongbuk region, limiting the generalizability of our findings. Second, as a secondary data analysis using reporting data from IDIS and epidemiological investigation results, the possibility of underreporting or data entry errors cannot be ruled out. Third, for some clusters, the number of cases was entered as 0 in the outbreak epidemiological investigation results, or the number of cases did not match the individual epidemiological investigation data, and thus were corrected based on the individual epidemiological investigation data. Some outbreaks for which case information could not be confirmed were excluded from the analysis of cluster size and outbreak duration. Fourth, the cause of cluster occurrence could not be directly evaluated because factors that could influence cluster occurrence, such as vaccination history, breakthrough infection status, school size, and number of classes, were not included. Fifth, because rates using the number of facilities or enrolled students by facility type as denominators were not calculated, the risk of cluster occurrence by facility type could not be compared.

In conclusion, analysis of the occurrence patterns and epidemiological characteristics of varicella outbreaks in the Gyeongbuk region during 2022–2025 demonstrated that outbreaks were most common in elementary schools, and outbreaks with a size of 11 or more cases also mainly occurred in elementary schools. Therefore, the results of this study can be utilized as evidence for reviewing school-based varicella prevention and control and future vaccination policies.

## Figures and Tables

**Figure 1:**
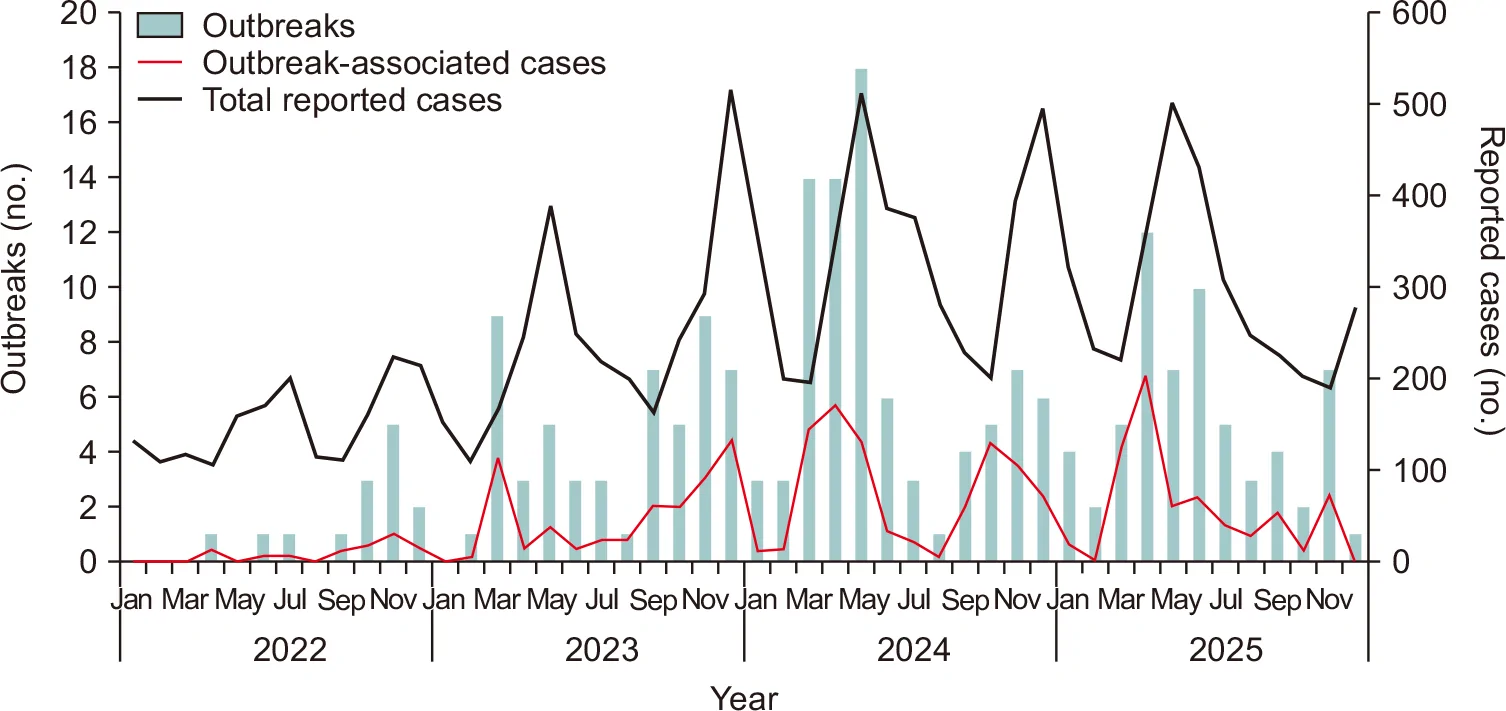
Monthly numbers of outbreaks, outbreak-associated cases, and total reported varicella cases in the Daegu–Gyeongbuk region, 2022–2025

**Table 1:** Selection of varicella outbreaks in the Daegu–Gyeongbuk region, 2022–2025

Category	2022	2023	2024	2025	Total
Initially identified outbreaks	14	53	95	73	235
Excluded outbreaks	0	0	11	11	22
Reclassified to the previous yeara)	0	0	2	0	2
Duplicate outbreak	0	0	0	1	1
Deleted by public health centers	0	0	8	6	14
Outside the study area	0	0	1	4	5
Outbreaks included in the study	14	53	84	62	213

Unit: outbreaks. ^a)^After review, these outbreaks were confirmed to have occurred in the previous year and were therefore reclassified to avoid duplicate counting.

**Table 2:** General characteristics of varicella outbreaks in the Daegu–Gyeongbuk region, 2022–2025

Characteristics	Category	2022	2023	2024	2025	Total
Region	Total	14 (100.0)	53 (100.0)	84 (100.0)	62 (100.0)	213 (100.0)
	Daegu	10 (71.4)	33 (62.3)	67 (79.8)	47 (75.8)	157 (73.7)
	Gyeongsangbuk-do	4 (28.6)	20 (37.7)	17 (20.2)	15 (24.2)	56 (26.3)
Facility type	Total	14 (100.0)	53 (100.0)	84 (100.0)	62 (100.0)	213 (100.0)
	Childcare center	1 (7.1)	1 (1.9)	3 (3.6)	4 (6.5)	9 (4.2)
	Kindergarten	0	3 (5.7)	6 (7.1)	0	9 (4.2)
	Elementary school	10 (71.4)	32 (60.4)	49 (58.3)	36 (58.1)	127 (59.6)
	Middle and high schools	3 (21.4)	16 (30.2)	24 (28.6)	15 (24.2)	58 (27.2)
	Othera)	0	1 (1.9)	2 (2.4)	7 (11.3)	10 (4.7)

Unit: n (%). ^a)^Other settings included a factory, an agricultural cooperative, a hospital, a church, a welfare facility, a rehabilitation center, and a military unit.

**Table 3:** Outbreak size and duration of varicella outbreaks by year and facility type in the Daegu–Gyeongbuk region, 2022–2025

Variable	2022 (n=14)	2023 (n=51)	2024 (n=84)	2025 (n=60)	Overalla) (n=209)	p-value
Outbreak size (no. of cases)	6 (3.5–8.0)	7 (4.5–12.5)	6 (3.0–15.0)	10 (4.0–13.3)	7 (3.0–14.0)	0.353
Range	2–15	2–61	2–62	2–43	2–62	
Outbreak duration (day)	23 (12.5–32.3)	32 (20.0–57.5)	23 (16.0–45.3)	34 (20.5–56.5)	31 (16.0–49.0)	0.010
Range	4–70	2–114	1–108	1–112	1–114	

Variable	Childcare centers (n=9)	Kindergartens (n=9)	Elementary schools (n=127)	Middle and high schools (n=57)	Other facilities (n=7)	p-value
Outbreak size (no. of cases)	6 (3.0–6.0)	11 (9.0–12.0)	8 (4.0–15.5)	6 (3.0–11.0)	3 (2.0–7.0)	0.022
Range	3–15	5–25	2–62	2–43	2–11	
Outbreak duration (day)	17 (6.0–31.0)	27 (20.0–35.0)	33 (16.0–58.0)	30 (18.0–49.0)	18 (7.0–18.5)	0.020
Range	2–32	7–80	1–112	2–114	1–34	

Values are presented as median (interquartile range) and range. Differences by year and facility type were analyzed using the Kruskal-Wallis test, with the Dwass-Steel-Critchlow-Fligner test used for post hoc comparisons when the overall test was statistically significant. ^a)^Two outbreaks with only one confirmed case and two outbreaks with unavailable patient information were excluded from the analysis.
