# Supplementary material for: Epidemiological Characteristics of Varicella Outbreaks in Daegu and Gyeongsangbuk-do, the Republic of Korea, 2022–2025
Source: Public Health Wkly Rep. 2026 Oct 22;19(40):1685–700. doi: 10.56786/PHWR.2026.19.40.1 (PMC13691639; doi:10.56786/PHWR.2026.19.40.1)
Supplement: Translation

# Translation: 2022–2025년 경북권 수두 집단사례의 역학적 특성

**Authors:** 성희 오, 민제 이, 숙현 김, 명재 황, 상연 조

**Affiliations:** 1 질병관리청 의료안전예방국 예방접종관리과; 2 질병관리청 경북권질병대응센터 감염병대응과

**Keywords:** 수두, 집단발생, 학교, 예방접종

## Abstract

**목적:**

2022–2025년 경북권(대구광역시, 경상북도) 수두 집단사례의 발생 양상과 역학적 특성을 기술하고자 하였다.

**방법:**

2022년 1월부터 2025년 12월까지 질병관리청 방역통합정보시스템에 보고된 경북권 수두 집단사례를 대상으로 하였다. 총 235개 집단 중 전년도 집단으로 재분류, 중복, 삭제 및 관할 외 사례를 제외한 213개 집단을 분석하였으며, 집단 규모와 유행기간은 환자 정보를 확인할 수 있는 209개 집단(2,253명)을 대상으로 분석하였다.

**결과:**

수두 집단사례는 2022년 14건에서 2024년 84건으로 증가하였으며, 집단 관련 환자 수도 2024년 898명으로 가장 많았다. 전체 집단의 59.6%가 초등학교에서 발생하였으며, 11명 이상 규모의 집단사례도 대부분 초등학교에서 발생하였다. 집단 규모는 연도별로 유의한 차이가 없었으나 유행기간은 연도별로 유의한 차이를 보였다.

**결론:**

2022–2025년 경북권 수두 집단사례의 발생 양상과 역학적 특성을 분석한 결과, 초등학교에서 집단사례가 가장 많았으며 11명 이상 규모의 집단사례도 주로 초등학교에서 발생하는 양상을 보였다. 본 연구 결과는 학교 중심의 수두 예방관리와 향후 예방접종 정책을 검토하기 위한 기초자료로 활용될 수 있을 것이다.

**핵심요약**

**① 이전에 알려진 내용은?**

2018–2020년 전국 수두 집단사례 분석에서 초등학교의 집단사례가 가장 많았으며, 집단 규모와 유행기간도 어린이집과 유치원보다 큰 것으로 보고되었다.

**② 새로이 알게 된 내용은?**

2022–2025년 경북권의 수두 집단사례는 2024년까지 증가한 후 2025년 감소하였으며, 집단 규모는 연도별로 유의한 차이가 없었으나 유행기간은 유의한 차이를 보였다.

**③ 시사점은?**

2022–2025년 경북권 수두 집단사례의 역학적 특성을 확인하였으며, 학교 중심의 예방관리와 향후 예방접종 정책을 검토하기 위한 기초자료로 활용될 수 있다.

## 서 론

수두는 수두-대상포진 바이러스(varicella-zoster virus)에 의한 급성 발진성 감염병으로 동물이나 곤충 등 다른 매개체 없이 사람에 의해서만 전파되는 감염병이다. 수두 환자의 수포성 병변에 직접 접촉하거나, 급성 수두 또는 대상포진 환자의 피부병변 수포액이나 호흡기 분비물로부터 발생한 에어로졸을 흡입하여 전파된다[1]. 전염성이 매우 높아 가족 내 수두 환자가 있을 경우 수두에 감수성이 있는 가족접촉자에게 2차 전파율이 80–90%로 보고되고 있으며, 전염기간은 발진 발생 1–2일 전부터 모든 병변에 가피가 생성될 때까지 지속된다[2]. 수두의 잠복기는 노출 후 10–21일이며, 평균 잠복기는 14–16일이다. 수두의 임상증상으로는 발열과 함께 반점(macules), 구진(papules), 수포(vesicles), 농포(pustules), 가피(crust) 순으로 24시간 이내에 빠르게 진행된다. 건강한 소아의 경우 증상이 경미하고 발진 시작 2–4일 이내에 전신증상이 호전되며, 면역력이 떨어지는 소아나 성인의 경우 발진 부위 2차 감염, 라이증후군, 뇌염, 폐렴 등 합병증 발생률이 더 높을 수 있다. 또한 임신 20주 이내 임신부가 원발성 수두에 감염된 경우 신생아 기형과 관련된 선천성 수두 증후군이 발생할 수 있다[1].

수두는 코로나바이러스감염증-19(코로나19) 대유행 이전 연간 약 8–9만 명의 환자가 신고되었으며, 학령기에 주로 발생하는 대표적인 법정감염병이다[3]. 또한 학기 중인 4–7월과 11–12월에 발생이 증가하는 계절적 특성을 나타내고 있다[1].

우리나라는 2005년부터 수두를 국가예방접종(National Immunization Program, NIP)에 포함하였고, 모든 소아를 대상으로 생후 12–15개월에 1회 접종을 권고하고 있다[4]. 2020–2022년 만 2세 소아의 수두 예방접종률은 96% 이상으로 보고되었으며[5-7], 수두 백신의 예방 효과는 1회 접종 시 약 79%로 보고되고 있다[8].

질병관리청 감염병 포털 자료에 따르면 수두, 유행성이하선염, 백일해 등 일부 호흡기 감염병의 경우 코로나19 대유행 기간 감소하였다가 2022년 이후 점차 증가하는 경향을 보이고 있다[9]. 2022년 이후 지역 단위 수두 집단사례의 규모ㆍ유행기간을 분석한 국내 연구는 제한적이다. 이에 2022년부터 2025년까지 방역통합정보시스템에 보고된 경북권(대구광역시, 경상북도, 이하 경북권)의 수두 집단사례 자료를 활용하여 코로나19 일상회복 과정에서 경북권 수두 집단사례의 발생 양상과 역학적 특성을 분석하였다.

## 방 법

### 1. 연구대상

2022년 1월부터 2025년 12월까지 질병관리청 방역통합정보시스템에 수두 환자 또는 의사환자로 신고된 사례를 대상으로 하였다. 수두의 진단 및 신고 기준에 따라 환자는 수두에 부합하는 임상증상과 진단 검사기준에 따라 감염이 확인된 사람으로 하였으며, 의사환자는 임상증상과 역학적 연관성을 고려하여 수두가 의심되나 진단 검사기준에 부합하는 검사 결과가 없는 사람으로 하였다[1]. 집단사례는 2022–2025년 질병관리청 「예방접종감염병 관리 지침」의 유행사례 기준을 적용하였다[10-13]. 유행사례는 3주 이내 같은 학급 또는 시설에 2명 이상의 환자 또는 의사환자가 발생하거나, 2명 이상이 시간적ㆍ공간적으로 밀접하게 연관된 경우로 하였으며, 이 중 역학조사 결과 집단으로 분류된 사례를 연구대상으로 선정하였다. 총 235개 집단 중 전년도 집단으로 재분류, 중복, 보건소 삭제 및 경북권 관할 외 사례 22개를 제외한 213개 집단을 분석하였다(표 1). 집단 규모 및 유행기간은 집단 관련 환자 정보를 확인할 수 있는 집단으로 분석대상을 제한하였으며, 환자가 1명만 확인된 집단 2건과 환자 정보를 확인할 수 없는 집단 2건을 제외한 총 209개 집단(집단 관련 환자 2,253명)을 분석하였다.

### 2. 자료수집 및 관리

질병관리청 방역통합정보시스템을 이용하여 2026년 1월 30일 기준으로 2022년부터 2025년까지 경북권에 신고된 수두 집단사례와 집단 관련 개별 역학조사 자료를 1차 수집하였다. 이후 2025년 신고자료의 최신화를 위해 2026년 7월 22일 기준으로 자료를 재수집하여 최종 자료를 구축하였다.

집단사례의 환자 수는 집단사례 역학조사 결과와 개별 역학조사 자료를 비교하여 확인하였으며, 두 자료 간 환자 수가 불일치하는 경우 개별 역학조사 자료를 기준으로 보정하였다. 중복 사례, 보건소 삭제 사례 및 경북권 관할 외 사례는 제외하였으며, 전년도 집단에 해당하는 사례는 최초 환자의 증상 발생일 기준으로 재분류하였다.

### 3. 변수정의

집단사례의 발생연도와 발생월은 집단 내 최초 환자의 증상 발생일을 기준으로 분류하였다. 발생지역은 집단이 발생한 시ㆍ도로 구분하였으며, 시설유형은 어린이집, 유치원, 초등학교, 중ㆍ고등학교 및 기타로 분류하였다. 기타에는 공장, 영농조합, 병원, 교회, 복지시설, 재활원 및 군부대를 포함하였다. 집단 규모는 집단 관련 환자 수로 정의하였다. 유행기간은 집단 내 최초 환자의 증상 발생일부터 마지막 환자의 증상 발생일까지로 하였으며, 최초 및 마지막 증상 발생일을 모두 포함하기 위해 두 날짜의 차이에 1일을 더하여 산정하였다.

### 4. 분석방법

집단 규모와 유행기간의 연도 및 시설유형별 차이는 Kruskal-Wallis 검정을 이용하여 분석하였으며, 통계적으로 유의할 경우 Dwass-Steel-Critchlow-Fligner 사후검정을 시행하였다. 통계분석은 Jamovi (ver. 2.3.28)를 이용하였으며, 자료 집계 및 정리, 그래프 작성은 Microsoft Excel 2016을 사용하였다. 통계적 유의수준은 p<0.05로 설정하였다.

### 5. 인공지능 도구 활용

본 원고의 일부 영문 번역 및 문장 교정에 인공지능 도구 ChatGPT (GPT-5.6, OpenAI)를 보조적으로 활용하였으며, 해당 내용은 저자가 검토하고 수정하였다.

## 결 과

### 1. 일반적 특성

2022년부터 2025년까지 경북권에서 확인된 수두 집단사례는 총 213건이며, 지역별로는 대구광역시 157건(73.7%), 경상북도 56건(26.3%)으로 대구광역시에서의 보고 비율이 높았다. 시설유형별로는 초등학교가 127건(59.6%)으로 가장 많았으며, 중ㆍ고등학교가 58건(27.2%), 기타 10건(4.7%), 어린이집과 유치원이 각각 9건(4.2%) 순이었다. 연도별로도 모든 연도에서 초등학교의 집단사례가 가장 많았다(표 2).

### 2. 연도별 및 월별 수두 집단사례 발생현황

연도별 수두 집단사례 건수는 2022년 14건, 2023년 53건, 2024년 84건, 2025년 62건이었으며, 2024년에 가장 많이 발생하였다. 집단 관련 환자 수는 2022년 97명, 2023년 572명, 2024년 898명, 2025년 686명이었다. 월별 집단 발생 건수가 가장 많은 월은 2022년 11월 5건, 2023년 3월 9건, 2024년 5월 18건, 2025년 4월 13건이었다. 월별 집단 관련 환자 수가 가장 많은 월은 2022년 11월 31명, 2023년 12월 132명, 2024년 4월 171명, 2025년 4월 201명이었다.

경북권 전체 수두 환자 수는 2023년부터 2025년까지 봄철과 연말에 증가하고 여름철에는 감소하는 경향을 보였다(그림 1).

### 3. 지역 및 시설유형별 발생현황

지역별 집단 관련 환자 수는 대구광역시와 경상북도 모두 2024년에 각각 664명과 234명으로 가장 많았다. 시설유형별로는 모든 연도에서 초등학교의 집단 관련 환자 수가 가장 많았으며, 2022년 74명, 2023년 409명, 2024년 589명, 2025년 457명이었다. 중ㆍ고등학교는 초등학교 다음으로 집단 관련 환자 수가 많았으며, 2024년에 203명으로 가장 많았다. 집단 규모별로는 초등학교가 11–20명 집단 49건 중 30건(61.2%), 21–40명 집단 23건 중 17건(73.9%), 41명 이상 집단 5건 중 4건(80.0%)을 차지하였다.

### 4. 집단 규모 및 유행기간

집단 규모 및 유행기간 분석 대상인 209개 집단(집단 관련 환자 2,253명)을 분석한 결과, 집단 규모별로는 2–10명 집단이 132건(63.2%)으로 가장 많았으며, 11–20명 49건(23.4%), 21–40명 23건(11.0%), 41명 이상 5건(2.4%) 순이었다. 연도별로는 2–10명 집단의 경우 2022년 11건, 2023년 31건, 2024년 55건, 2025년 35건이었으며, 11–20명 규모의 집단은 각각 3건, 12건, 17건, 17건이었다. 41명 이상 집단의 경우 2023년 1건, 2024년 3건, 2025년 1건이었으며, 2022년에는 발생하지 않았다.

집단 규모의 중앙값은 전체 7명(3.0–14.0명)이었으며, 연도별로는 2022년 6명(3.5–8.0명), 2023년 7명(4.5–12.5명), 2024년 6명(3.0–15.0명), 2025년 10명(4.0–13.3명)으로 2025년에 가장 컸으나, 연도별 집단 규모의 차이는 통계적으로 유의하지 않았다(p=0.353).

2022–2025년 전체 유행기간의 중앙값은 31일(16.0–49.0일)이었으며, 2022년 23일(12.5–32.3일), 2023년 32일(20.0–57.5일), 2024년 23일(16.0–45.3일), 2025년 34일(20.5–56.5일)로 2025년이 가장 길었다. 또한, 연도별 유행기간은 통계적으로 유의한 차이가 있었다(p=0.010) (표 3). 사후검정 결과, 2025년의 유행기간은 2022년(p=0.040)과 2024년(p=0.038)에 비해 유의하게 길었던 것을 확인하였으나, 그 외 연도 간에는 유의한 차이가 없었다.

시설유형별 집단 규모의 중앙값은 어린이집 6명(3.0–6.0명), 유치원 11명(9.0–12.0명), 초등학교 8명(4.0–15.5명), 중ㆍ고등학교 6명(3.0–11.0명), 기타 3명(2.0–7.0명)이었다(표 3). 시설유형별 집단 규모는 통계적으로 유의한 차이가 있었으나(p=0.022) 사후검정 결과, 개별 시설유형 간 유의한 차이가 없었다. 유행기간의 중앙값은 어린이집 17일(6.0–31.0일), 유치원 27일(20.0–35.0일), 초등학교 33일(16.0–58.0일), 중ㆍ고등학교 30일(18.0–49.0일), 기타 18일(7.0–18.5일)이었다. 시설유형별 유행기간은 통계적으로 유의한 차이가 있었으나(p=0.020), 사후검정 결과 개별 시설유형 간 유의한 차이가 없었다.

## 논 의

이 연구는 2022년부터 2025년까지 경북권에 보고된 수두 집단사례 213건을 분석하였다. 수두 집단사례는 2022년부터 2024년까지 증가하여 2024년에 84건으로 가장 많았으며, 2025년에는 62건으로 감소하였다. 집단 관련 환자 수도 2022년부터 2024년까지 증가하여 2024년에 898명으로 가장 많았으며, 2025년에는 686명으로 감소하였다. 선행연구에서는 코로나19 대유행 기간 감소하였던 수두 발생이 사회적 거리두기 해제와 대면활동 재개 이후 다시 증가한 것으로 보고된 바 있다[14,15]. 다만 본 연구는 코로나19 대유행 이전의 자료를 포함하지 않아 대유행 전후 발생 양상을 직접 비교하는 데에는 한계가 있었으며, 2025년에는 집단사례와 집단 관련 환자 수가 모두 전년도보다 감소하였으나 연구기간이 4년으로 짧아 이러한 감소가 지속적인 추세인지 판단하기 어렵다.

시설유형별로는 초등학교가 전체 집단의 59.6%로 가장 많았으며, 집단 발생 환자 수 또한 모든 연도에서 가장 큰 비중을 차지했다. 코로나19 대유행 이전 기간이 포함된 2018–2020년 수두 집단사례 분석에서도 초등학교가 전체 집단의 59.3%로 가장 높은 비중을 차지하여 본 연구 결과와 유사한 양상을 보였다. 또한 초등학교는 학급의 밀도가 상대적으로 높고, 방과 후 활동 등에 따른 학년 및 학급 간 집단발생에 영향을 미칠 가능성이 제시된 바 있다[2].

집단 규모별로는 11–20명 규모 61.2%, 21–40명 규모 73.9%, 41명 이상 규모 80.0%가 초등학교에서 발생하였다. 시설유형별로 집단 규모와 유행기간을 비교한 결과, 두 지표 모두 통계적으로 유의한 차이가 있었으나, 사후검정에서는 개별 시설유형 간 유의한 차이가 없었다. 집단 규모의 중앙값은 연도별로 유의한 차이가 없었으나, 유행기간의 중앙값은 연도에 따라 유의한 차이를 보였다. 사후검정 결과 2025년의 유행기간은 2022년과 2024년에 비해 유의하게 길었다. 국내 선행연구에서도 수두 집단사례의 유행기간 중앙값이 연도에 따라 차이를 보인 바 있다[16]. 다만 선행연구에서도 이러한 차이의 원인은 명확히 제시되지 않았다. 본 연구에서도 유행기간의 연도별 차이에 영향을 미치는 요인을 확인하기 어려웠으며, 향후 충분한 연도별 자료가 축적되면 관련 요인을 추가로 분석해 볼 필요가 있다.

현재 우리나라는 생후 12–15개월 소아를 대상으로 NIP의 일환으로 수두 예방접종을 1회 시행하고 있다[4]. 이번 연구에서는 초등학교를 중심으로 집단사례가 지속적으로 발생하고, 비교적 큰 규모의 사례도 확인되었다. 기존 연구들에 따르면 수두 백신의 예방 효과는 1회 접종 시 약 79%에 불과하나, 2회 접종 시 약 94%로 상승하는 것으로 보고된 바 있다[8]. 초등학생 연령대에서의 수두 발생 증가가 시간 경과에 따른 백신 효과의 감소(waning immunity)에 기인한 것인지[17], 혹은 1차 기본 접종 누락이나 돌파감염(breakthrough infection)의 증가로 인한 것인지 파악하는 것이 중요하다. 이를 위해 향후 연구에서는 연령별 수두 백신 접종 완료율과 돌파감염 비율을 연계하여 분석할 필요가 있다[17,18].

그러나 본 연구에서는 몇 가지 제한점이 있다. 첫째, 연구 대상을 경북권으로 한정하여 연구 결과를 전국으로 일반화하는 데 한계가 있다. 둘째, 방역통합정보시스템 신고자료와 역학조사 결과를 이용한 이차자료 분석으로 신고 누락이나 정보 입력 오류의 가능성을 배제할 수 없다. 셋째, 일부 집단은 집단사례 역학조사 결과에 환자 수가 0명으로 입력되어 있거나 개별 역학조사 자료와 환자 수가 일치하지 않아 개별 역학조사 자료를 기준으로 보정하였으며, 환자 정보를 확인할 수 없는 일부 집단사례는 집단 규모와 유행기간 분석에서 제외하였다. 넷째, 예방접종력, 돌파감염 여부, 학교 규모 및 학급 수 등 집단발생에 영향을 미칠 수 있는 요인을 포함하지 못하여 발생원인을 직접 평가하지 못하였다. 다섯째, 시설유형별 시설 수 또는 재학생 수를 분모로 한 발생률을 산출하지 않아 시설유형별 집단발생 위험을 직접 비교하는 데 한계가 있다.

결론적으로 2022–2025년 경북권 수두 집단사례의 발생 양상과 역학적 특성을 분석하여 초등학교에서 집단사례가 가장 많고 11명 이상 규모의 집단사례도 주로 초등학교에서 발생하는 양상을 확인하였다. 따라서 본 연구 결과는 학교 중심의 수두 예방관리와 향후 예방접종 정책을 검토하기 위한 기초자료로 활용될 수 있을 것이다.

## Figures and Tables

**그림 1:** 2022–2025년 경북권의 월별 수두 집단사례 건수, 집단 관련 환자 수 및 전체 신고 환자 수

**표 1:** 2022–2025년 경북권 수두 집단사례의 연구대상 선정

구분	2022년	2023년	2024년	2025년	전체
원자료 집단	14	53	95	73	235
제외 집단	0	0	11	11	22
전년도 집단으로 재분류a)	0	0	2	0	2
중복	0	0	0	1	1
보건소 삭제	0	0	8	6	14
관할 외	0	0	1	4	5
최종 집단	14	53	84	62	213

단위: 건. ^a)^검토 결과 전년도 발생 집단에 해당하는 것으로 확인되어 중복 집계를 방지하기 위해 전년도 집단으로 재분류.

**표 2:** 2022–2025년 경북권 수두 집단사례의 일반적 특성

특성	구분	2022년	2023년	2024년	2025년	전체
지역	계	14 (100.0)	53 (100.0)	84 (100.0)	62 (100.0)	213 (100.0)
	대구광역시	10 (71.4)	33 (62.3)	67 (79.8)	47 (75.8)	157 (73.7)
	경상북도	4 (28.6)	20 (37.7)	17 (20.2)	15 (24.2)	56 (26.3)
시설유형	계	14 (100.0)	53 (100.0)	84 (100.0)	62 (100.0)	213 (100.0)
	어린이집	1 (7.1)	1 (1.9)	3 (3.6)	4 (6.5)	9 (4.2)
	유치원	0	3 (5.7)	6 (7.1)	0	9 (4.2)
	초등학교	10 (71.4)	32 (60.4)	49 (58.3)	36 (58.1)	127 (59.6)
	중ㆍ고등학교	3 (21.4)	16 (30.2)	24 (28.6)	15 (24.2)	58 (27.2)
	기타a)	0	1 (1.9)	2 (2.4)	7 (11.3)	10 (4.7)

단위: 건(%). ^a)^기타: 공장, 영농조합, 병원, 교회, 복지시설, 재활원, 군부대 포함.

**표 3:** 2022–2025년 경북권 수두 집단사례의 연도 및 시설유형별 집단 규모와 유행기간

특성	2022년(n=14)	2023년(n=51)	2024년(n=84)	2025년(n=60)	전체a) (n=209)	p-value
집단 규모(명)	6 (3.5–8.0)	7 (4.5–12.5)	6 (3.0–15.0)	10 (4.0–13.3)	7 (3.0–14.0)	0.353
최소–최대	2–15	2–61	2–62	2–43	2–62	
유행기간(일)	23 (12.5–32.3)	32 (20.0–57.5)	23 (16.0–45.3)	34 (20.5–56.5)	31 (16.0–49.0)	0.010
최소–최대	4–70	2–114	1–108	1–112	1–114	

특성	어린이집(n=9)	유치원(n=9)	초등학교(n=127)	중·고등학교(n=57)	기타(n=7)	p-value
집단 규모(명)	6 (3.0–6.0)	11 (9.0–12.0)	8 (4.0–15.5)	6 (3.0–11.0)	3 (2.0–7.0)	0.022
최소–최대	3–15	5–25	2–62	2–43	2–11	
유행기간(일)	17 (6.0–31.0)	27 (20.0–35.0)	33 (16.0–58.0)	30 (18.0–49.0)	18 (7.0–18.5)	0.020
최소–최대	2–32	7–80	1–112	2–114	1–34	

값은 중앙값(사분위범위)과 최소–최대로 제시. 연도별 및 시설유형별 차이는 Kruskal-Wallis 검정으로 분석하였으며, 통계적으로 유의한 경우 Dwass-Steel-Critchlow-Fligner 사후검정을 시행. ^a)^환자가 1명만 확인된 집단 2건과 환자 정보를 확인할 수 없는 집단 2건 분석 제외.
